# The pathogenesis and surgical treatment progress of gastroesophageal reflux disease

**DOI:** 10.3389/fsurg.2026.1846228

**Published:** 2026-05-25

**Authors:** Longmiao Gu, Fan Wu, Jie Cheng, Xiaojun Shen

**Affiliations:** 1 Gusu School, Nanjing Medical University, The First People’s Hospital of Kunshan, Suzhou, Jiangsu, China; 2 The First People’s Hospital of Kunshan, Affiliated Kunshan Hospital to Jiangsu University, Suzhou, Jiangsu, China

**Keywords:** gastroesophageal reflux disease, pathogenesis, surgical treatment, hiatal hernia, fundoplication

## Abstract

Gastroesophageal reflux disease is a common gastrointestinal disorder worldwide, with a particularly high prevalence in Western countries, and its incidence has been rising steadily in Asia in recent years. The pathogenesis involves dysfunction of the lower esophageal sphincter, inadequate support of the crural diaphragm, disruption of the His angle and gastroesophageal flap valve, the formation of a postprandial acid pocket, and delayed gastric emptying in some patients. Surgical intervention is required for patients with severe symptoms or complications. Traditional fundoplication provides effective symptom control, but postoperative complications such as dysphagia and gas-bloat syndrome remain concerns; in cases of short esophagus or large hiatal hernia, Collis gastroplasty or mesh reinforcement may be necessary. In recent years, the advancement of minimally invasive techniques has facilitated the application of robot-assisted surgery, magnetic sphincter augmentation, transoral incisionless fundoplication, and lower esophageal sphincter electrical stimulation, which emphasize minimal invasiveness, functional preservation, and individualized approaches. Whether the concept of “asymptomatic elective repair”, as applied in inguinal hernia, can be extended to certain hiatal hernia patients remains to be further explored.

## 1 Introduction

Gastroesophageal reflux disease (GERD) is a common gastrointestinal disorder characterized by the reflux of gastric contents into the esophagus, leading to symptoms such as retrosternal burning, regurgitation, and dysphagia (1). The prevalence of GERD has increased in parallel with changes in lifestyle and dietary habits, exerting a considerable impact on patients' quality of life. Multiple epidemiological studies have demonstrated a rising global prevalence, with an overall rate of approximately 14%, though substantial regional variation exists: North America shows the highest prevalence (19.6%), followed by Europe (14.1%), Latin America and the Caribbean (12.9%), and Asia (12.9%) (2). In China, the prevalence is estimated at around 10%. By 2035, the number of affected individuals aged 15–49 years is projected to reach 530 million, with a continued upward trend (3). The higher prevalence in North America and Europe compared with Asia can be attributed to multiple factors: (1) dietary and lifestyle differences, as Western diets are typically rich in high-protein and high-fat foods such as fried dishes and cheese, which delay gastric emptying and reduce lower esophageal sphincter (LES) pressure, thereby promoting reflux (4, 5) (2) excessive fat intake contributing to overweight and obesity (6), with obesity recognized as an independent risk factor that increases intra-abdominal pressure and induces LES relaxation (7); (3) Helicobacter pylori infection, which reduces gastric acid secretion due to glandular atrophy, thus lowering GERD risk (8). Notably, regions with high infection rates in Asia report lower GERD prevalence, but with increasing H. pylori eradication, GERD incidence has risen (9); (4) ethnic and genetic background, as lower prevalence in Asian populations may be associated with physiological differences such as acid secretion, esophageal length, and genetic susceptibility (10); (5) the cultural and cognitive differences, as variations in symptom description across cultures affect the accuracy of epidemiological assessments.

Although pharmacological therapy is effective in the majority of patients, surgical treatment becomes necessary for those with refractory disease or severe complications. The primary objective of surgery is to address the underlying cause of reflux. This review summarizes the current understanding of the pathogenesis of GERD and recent advances in surgical treatment, including novel techniques, outcome evaluation, and long-term prognosis.

## 2 Pathogenesis of GERD

The pathogenesis of GERD is multifactorial, mainly involving impaired antireflux mechanisms, reduced esophageal clearance, delayed gastric emptying, weakened esophageal mucosal defense, and obesity. The esophagogastric junction (EGJ) serves as the critical antireflux barrier and consists of the lower esophageal sphincter (LES), crural diaphragm (CD), phrenoesophageal ligament, and the His angle. Anatomical abnormalities occur when hiatal hernia (HH) develops, in which abdominal organs or tissues other than the esophagus protrude into the thoracic cavity through the widened esophageal hiatus. A substantial proportion of HH patients concurrently present with GERD. Among EGJ components, the LES is recognized as the most crucial structure, as its function directly influences the occurrence of reflux (11). Classification of hiatal hernia largely depends on whether the LES is displaced; for example, types II and III are differentiated by the position of the LES relative to the hiatus (12). Bonavina et al. first demonstrated in a classical experimental study that LES competency requires three structural conditions: adequate total length (2 cm), sufficient intra-abdominal length (1 cm), and adequate sphincter pressure (6 mmHg) (13). Deficiency in any of these significantly increases reflux risk, indicating that LES function depends on the interplay of these three factors (14). In recent years, transient lower esophageal sphincter relaxation (TLESR) has also been considered the predominant mechanism of GERD (15).

The crural diaphragm (CD) is another essential component of the antireflux barrier, and its dysfunction has been strongly associated with GERD. Using high-resolution manometry, Pandolfino et al. observed increased LES–CD separation and reduced inspiratory EGJ pressure in GERD patients, suggesting impaired CD function (16). Shafik proposed the concept of the “straining–crural reflex,” whereby sudden increases in intra-abdominal pressure induce reflexive contraction of the CD, thereby augmenting LES pressure and maintaining the integrity of the distal esophagus (17). Zdrhova et al. reported that targeted breathing exercises can strengthen diaphragmatic function and improve EGJ competence against reflux (18). Joshua et al. suggested that the high prevalence of GERD in patients with chronic pulmonary disease may be related to selective CD dysfunction (19). Mittal and colleagues systematically analyzed the structural and physiological synergy among the LES, CD, and phrenoesophageal ligament, forming a composite “coincide” structure that serves as an integrated barrier against reflux (20, 21). Disruption of this anatomical complex, such as when the LES separates from the CD, alters pressure distribution and results in loss of EGJ competence (22). The frequent coexistence of reflux symptoms in patients with HH further supports this concept. Andrews et al. (23) noted that CD contraction not only provides extrinsic support to the LES but also helps maintain the sharpness of the His angle through mechanical traction. When the CD relaxes, the His angle opens, allowing belching, reflux, and vomiting, a mechanism functionally analogous to the role of the puborectalis muscle in defecation control.

The His angle refers to the acute angle formed between the gastric fundus and the esophagus, which appears as a protruding mucosal fold on the lesser curvature under endoscopy, termed the gastroesophageal flap valve (GEFV). It represents a supplementary antireflux mechanism at the distal esophagus, and both structures essentially reflect the same anatomy viewed from different perspectives (24). Alterations in the His angle are often associated with the occurrence and severity of GERD. Studies have shown that blunting or laxity of the His angle frequently leads to GEFV distortion and significantly increases reflux risk (25). Fundoplication achieves its antireflux effect partly by reconstructing the GEFV. Hill and colleagues confirmed the existence of the GEFV through cadaveric dissection and *in vivo* endoscopic observation, and established a four-grade endoscopic classification system (Hill classification), which more effectively predicts the presence and severity of reflux (26).

In addition, the acid pocket plays an important role in the pathogenesis of GERD. Fletcher et al. first introduced the concept of the “acid pocket” using dual pH-probe monitoring, and both *in vivo* and *in vitro* studies confirmed that postprandial gastric juice near the squamocolumnar junction is not effectively buffered by food, forming an independent acid pocket that is located directly at, or even migrating above, the gastroesophageal junction (27). This provides the anatomical basis for reflux symptoms and esophageal mucosal injury. Kahrilas et al. demonstrated that acid pockets in GERD patients are more likely to migrate into the esophagus, especially in the supine position, thereby increasing reflux risk (28). Wu et al. further showed that acid pockets in GERD patients are more persistent, more acidic, and longer in extent, suggesting that the acid pocket may serve as a therapeutic target (29). Jo and Rohof et al. revealed that proton pump inhibitors (PPIs) and alginate-based antacids can regulate or displace the acid pocket, providing novel therapeutic approaches for GERD (30, 31).

In addition to impaired antireflux structures, delayed gastric emptying may also contribute in certain patients. Gastric emptying delay leads to gastric content retention, increased intragastric pressure, and transient lower esophageal sphincter relaxation, thereby inducing reflux (4). Fass et al. noted that although delayed gastric emptying is common among GERD patients, particularly in obese or diabetic populations, its correlation with reflux frequency or symptom severity remains inconclusive, and the causal relationship is still under debate (32). Tavakkoli et al. reported that among patients refractory to proton pump inhibitors, the frequency and duration of reflux episodes were not significantly different between those with impaired gastric emptying and those with normal emptying (33). This suggests that delayed gastric emptying may not represent the primary mechanism of reflux but rather a potential cofactor contributing to symptom exacerbation. Therefore, assessment of gastric motility may be considered preoperatively but should not serve as a core determinant in surgical decision-making.

These mechanisms are often intertwined, resulting in heterogeneity in the symptoms and severity of GERD. Management typically requires comprehensive evaluation of these factors, with individualized strategies including lifestyle modification, pharmacological therapy, and, in selected cases, surgical intervention.

## 3 Surgical treatment

Surgical treatment is not considered a first-line approach for GERD, while there are clear indications that exist for certain patients. With increasing understanding of the disease, surgical indications have evolved. Currently, surgery is primarily recommended for patients who fail medical therapy, present with structural abnormalities, or develop severe complications. Expanding evidence has also broadened the scope of candidates. Common surgical indications include: (1) long-term, regular use of proton pump inhibitors with unsatisfactory efficacy or intolerance to adverse effects (34, 35); (2) effective pharmacological therapy but requiring high-dose, long-term maintenance, in patients strongly preferring surgery (36); (3) coexisting severe complications such as Los Angeles grade C or D esophagitis or Barrett's esophagus (37, 38); (4) predominant extraesophageal manifestations, particularly severe or life-threatening symptoms such as laryngospasm and asthma (39); and (5) significant anatomical abnormalities, such as hiatal hernia or GEFV graded III–IV (40, 41). Preoperative assessment should include high-resolution manometry, 24-h esophageal pH-impedance monitoring, upper endoscopy, and barium swallow, to comprehensively evaluate mucosal injury, anatomical abnormalities, and gastrointestinal motility, thereby guiding optimal surgical selection (42–45).

With the deepening understanding of GERD pathogenesis, the focus of antireflux surgery has also shifted. According to the surgical emphasis, procedures can be broadly divided into three categories. The first category is conventional antireflux reconstruction, represented by Nissen (360°) fundoplication, Toupet (270°) fundoplication, and Dor (180°) fundoplication. These procedures increase the intra-abdominal length and pressure of the LES, while simultaneously repairing hiatal hernia, restoring crural diaphragm function, and reconstructing the His angle, thereby re-establishing the integrity of the EGJ complex. This allows for both functional restoration and structural reconstruction, effectively preventing reflux. The second category addresses specific anatomical conditions, such as large hiatal hernia or short esophagus. In these cases, Collis gastroplasty or mesh reinforcement may be added on the basis of standard fundoplication. Mesh can reinforce the diaphragm, but current high-quality evidence is lacking to support its routine use, and mesh-related complications should be carefully considered. The third category comprises novel functional enhancement procedures, including magnetic sphincter augmentation (LINX, MSA), transoral incisionless fundoplication (TIF), and LES electrical stimulation (EndoStim). These approaches provide the advantages of being minimally invasive, function-preserving, and reversible, thereby expanding surgical options.

The issue of short esophagus is an important challenge encountered during surgery for GERD or large hiatal hernia, and it can be classified as true or false. True short esophagus results from chronic inflammation and fibrotic contraction leading to anatomical shortening, whereas false short esophagus is usually due to large hiatal hernia or upward migration of the EGJ (46). Gastal et al. reported that preoperative diagnosis of short esophagus is difficult, and intraoperative assessment remains the gold standard. The “tension test” performed after thorough mediastinal mobilization is considered definitive, and esophageal stricture is the most important predictor of true short esophagus, often indicating the need for additional Collis gastroplasty (47).

In the repair of large or recurrent hiatal hernia, the crural diaphragm often contains limited tendinous tissue, and simple suture repair may result in a higher recurrence rate due to tissue fragility or excessive tension. To enhance repair strength and reduce suture–muscle tension, mesh reinforcement has been increasingly applied in clinical practice. Commonly used materials include permanent synthetic meshes (e.g., polypropylene), biologic meshes (e.g., porcine pericardium), and absorbable synthetic meshes. However, mesh-related complications such as migration, seroma, infection, and esophageal erosion must be carefully considered. Given these safety concerns, many surgeons currently prefer absorbable meshes (48). Analatos et al. conducted a randomized controlled trial with 106 patients and a mean follow-up of 13 years, reporting recurrence rates of 38% in the mesh group vs. 31% in the suture-only group, with no significant difference. These findings do not support the routine use of mesh (49). Future studies with larger sample sizes, multicenter participation, and long-term follow-up are required to validate the indications and establish standardized criteria for mesh application.

## 4 Complications and outcomes

Although surgery can relieve symptoms, postoperative complications remain a concern. The most common complications include dysphagia, recurrence, mesh-related problems, and gas-bloat syndrome (GBS). Dysphagia, often accompanied by belching, is one of the most frequent complications after hiatal hernia repair with fundoplication. It usually occurs in the early postoperative period, often transient and associated with local edema, and gradually improves with dietary adjustment within four weeks. However, a minority of patients develop severe or persistent dysphagia in the early or late postoperative period, requiring further evaluation with upper gastrointestinal radiography or endoscopy, and in some cases endoscopic dilation or reoperation (50). Long-term follow-up studies have shown dysphagia rates of 20%–45% after Nissen fundoplication, whereas the incidence is relatively lower after Toupet fundoplication (51, 52). Gas-bloat syndrome is also common, presenting as abdominal distension, inability to belch, postprandial fullness, and nausea. Although its mechanism is not fully understood, it is generally thought to result from impaired relaxation of the reconstructed GEFV, preventing gastric gas from entering the esophagus (53). It is more frequently observed after Nissen compared with partial fundoplication (54). Some studies have reported reoperation rates of approximately 7% at 10 years following laparoscopic surgery (55), underscoring the importance of long-term outcomes. Overall, the available evidence suggests that complication risk may be mitigated by structured preoperative physiological assessment, including reflux testing and high-resolution manometry, tailoring the operative strategy to esophagogastric junction anatomy and esophageal motility, and implementing standardized postoperative surveillance and management.

With increasing recognition of the shared pathophysiology between GERD and hiatal hernia, the timing of surgical referral has become an increasingly important clinical issue. In real-world practice, many older patients have a long history of hiatal hernia documented on prior examinations, yet receive no targeted management until symptoms worsen or complications develop. Such delay may permit gradual hernia enlargement and progressive disruption of the esophagogastric junction, resulting in large hernias at the time of surgery and potentially greater technical difficulty. This observation prompts a hypothesis that warrants careful evaluation: in selected patients with mild or minimally symptomatic hiatal hernia, an elective strategy analogous to asymptomatic repair in inguinal hernia might reduce progression and downstream surgical complexity. Nevertheless, current evidence is insufficient to support a specific early-intervention threshold. Prospective studies should therefore apply phenotype-based risk stratification using objective reflux metrics, esophagogastric junction morphology, and esophageal motility to determine who benefits from earlier intervention and to quantify the trade-off between symptom control and procedure-related adverse events.

## 5 Conclusion

GERD is a heterogeneous disorder driven by multiple interacting mechanisms, including impairment of the esophagogastric junction barrier (LES–crural diaphragm–flap valve complex), reflux triggers such as transient LES relaxations, refluxate characteristics and variability in esophageal clearance and mucosal susceptibility. Antireflux surgery aims to restore esophagogastric junction competence and should be considered in appropriately selected patients based on objective reflux testing, anatomical assessment, and esophageal motility. Fundoplication with hiatal repair remains the established surgical foundation, while adjunctive strategies may be required in specific anatomical settings. Emerging approaches such as magnetic sphincter augmentation, transoral incisionless fundoplication, and LES electrical stimulation broaden therapeutic options, but their use should remain phenotype-driven and supported by long-term comparative outcomes. Future research should prioritize standardized phenotyping, durable outcome reporting, and prospective studies to refine patient selection and determine the optimal timing of intervention.
